# Tuning the infrared resonance of thermal emission from metasurfaces working in near-infrared

**DOI:** 10.1038/s41598-023-34741-4

**Published:** 2023-05-09

**Authors:** Oana Rasoga, Daniela Dragoman, Adrian Dinescu, Christopher Andrew Dirdal, Irina Zgura, Florin Nastase, Angela Mihaela Baracu, Sorina Iftimie, Aurelian Catalin Galca

**Affiliations:** 1 National Institute of Materials Physics, 405A Atomistilor Street, 077125 Magurele, Romania; 2 Faculty of Physics, University of Bucharest, 405 Atomistilor Street, P.O. Box MG-11, 077125 Magurele, Romania; 3 National Institute for Research and Development in Microtechnologies-IMT Bucharest, 126A, Erou Iancu Nicolae Street, 077190 Voluntari, Romania; 4 SINTEF Microsystems and Nanotechnology, Gaustadalleen 23C, 0737 Oslo, Norway

**Keywords:** Materials science, Nanoscience and technology, Optics and photonics, Physics

## Abstract

We simulated numerically and demonstrated experimentally that the thermal emittance of a metasurface consisting of an array of rectangular metallic meta-atoms patterned on a layered periodic dielectric structure grown on top of a metallic layer can be tuned by changing several parameters. The resonance frequency, designed to be in the near-infrared spectral region, can be tuned by modifying the number of dielectric periods, and the polarization and incidence angle of the incoming radiation. In addition, the absorbance/emittance value at the resonant wavelength can be tuned by modifying the orientation of meta-atoms with respect to the illumination direction.

## Introduction

Nowadays, the production of plastics has exponentially increased since these inexpensive, lightweight, and durable materials^1^ can be used in a variety of industries. At the moment, only Europe produces 58 million tons of plastic, thus generating 25 million waste tons, from which only 30% is collected for recycling and 39% incinerated, the rest of 31% ending up in landfills and creating environmental problems^2,3^. Globally, according to the Organisation for Economic Co-operation and Development (OECD), the percentage of recycled waste is much smaller, of just 9%^4^. In consequence, the Plastic Strategy adopted recently by the European Commission pointed out that there is an urgent need of a “New Plastic Economy” based on the principles of the circular economy: reduce, reuse and recycle^5,6^. Thus, plastics and products based on plastics have to be designed to allow greater durability, reuse and high-quality recycling^5^. The first axis of this strategy, consisting of “improving the economics and the quality of plastic recycling”, imposes as mandatory the development of new simplified and low cost methods to sort different types of plastics in order to increase not just the quantity but also the quality of recycling^7^. However, the interest in recycling is justified as long as during the process the materials retain their original properties. In particular, in the case of plastics one has to be capable to separate at least the five major resins—polyethylene terephthalate (PET, (C_10_H_8_O_4_)_n_), polyvinyl chloride (PVC, (C2H3Cl)n), polypropylene (PP, [CH_2_CH(CH_3_)]_n_), polyethylene (PE, H(CH_2_CH_2_)_n_H) and polystyrene (PS, [CH_2_CH(C_6_H_5_)]_n_).

Usually, the waste disposal companies use not only one but a diversity of methods for sorting the materials. The most common method remains the manual sorting in combination with trammel separator, Eddi-current, induction sorting, gravimetric methods, X-ray technology, capacitance proximity or near infrared (NIR) spectroscopy^8–10^. Regarding the latter method, due to the fast evolution of sensor technologies based on light-matter interactions, the transfer from the bulk and heavy optical components to their small and lightweight counterparts, which preserve the optical performances and even provide new functionalities, led to the development of cost-effective NIR plastic identification systems operating at room temperature^11^. For example, the optical spectroscopic sensors specialized in the characterization of biological materials, pharmaceutics, nutrients, but also for plastic recycling in the near-infrared region, e.g., 0.8–2.5 µm range, enable probing of molecules containing C–H, N–H, S–H, and O–H bonds in a non-invasive manner^12^. This specific application is only one among others for which it is essential to develop novel sources that control the emission spectrum as well as the directionality of emitted light.

Conventional thermal sources are characterized by losses due to convection, conduction and emission in unwanted frequencies and directions. Whether the losses can be reduced or even eliminated by working under vacuum and choosing a proper design, various approaches have been devised for the control of emission, including subwavelength structures (metasurfaces) for generating directional or narrow-band thermal sources^13–16^.

Metasurfaces are the two-dimensional counterpart of three-dimensional metamaterials, which are subwavelength metallic/dielectric engineered structures with specific properties that are not observed in naturally occurring materials. Metasurfaces, as thin planar surfaces upon which periodic, quasi- or aperiodic meta-atoms arrays with different geometries and subwavelength spatial resolution are patterned, are easier to fabricate and exhibit superior wavefront modulation of the incident electromagnetic fields within the subwavelength scale^17–20^ compared to metamaterials, displaying at the same time lower losses and weaker dispersion.

Thermal emitters based on metasurfaces have been designed to operate from midinfrared (mid IR) to visible (VIS) spectral ranges^13,21–28^, but of interest for plastic waste disposal applications are NIR sources, because the principal categories of plastic have fingerprints in the 0.9–1.7 µm spectral range. Moreover, if portable devices are to be used for plastic detection and sorting, NIR sources integrated with optical elements, such as lenses, or even CMOS-compatible for integrated nanophotonic circuits, are desirable. In this paper we demonstrate, by numerical simulations and experiments, a metasurface with enhanced emissivity in the NIR spectrum that can be used in plastic waste disposal and can be fabricated with cost-effective technologies. The metasurface is formed from a layered dielectric periodic structure grown on top of a bottom metallic layer, and an array of rectangular metallic meta-atoms is then patterned on top of it. The resonance wavelength of the metasurface can be tuned by choosing the number of dielectric periods, while the value of the reflectance/absorbance at resonance can be tuned by changing the orientation of the meta-atoms.

Ideally, a thermal emitter should be highly-directional and operate in an application-defined range of frequencies. For plastic detection purposes, the bandwidth of the NIR source should not be very narrow since it has to accommodate fingerprints of several types of plastics. Therefore, generating highly directive thermal emission via narrow-band sources with increased temporal coherence and enhanced spatial coherence^29^ is not an option in our case. Directivity of thermal emitters can be achieved also by in- and out-coupling radiation to surface plasmon polaritons^24,27,30^, using epsilon-near-zero materials^13,31^, or by splitting the metasurface in an array of individually-controlled pixels^23^, for instance.

The design of the NIR thermal emitter presented in this paper is based on a multifunctional metasurface architecture^20^ that allows focusing of thermal emission and thus enhances the directivity of sources with not very narrow bandwidths. More precisely, the proposed metasurface is designed to act as a NIR source for absorption determination from NIR transmission measurements through various plastics. The determined absorption spectrum is then used to identify the plastic type. According to^20^, once the suitable metasurface for NIR absorption is found, it can be turned into a convergent thermal source, i.e., a NIR-emitting metalens that is significantly more directional than conventional thermal sources, if the meta-atoms are suitably rotated to implement the lens functionality; the rotation of meta-atoms does not influence the spectral response. Thus, the aim of this paper is to design, fabricate and characterize a metasurface with a suitable emissivity spectrum in NIR, which should not be very narrow in order to incorporate the wavelength regions of interest for various plastics.

According to Kirchhoff’s law, at thermodynamic equilibrium, the emissivity $\epsilon \left(\lambda ,\theta \right)$ of a source at a given wavelength (*λ*) and along a certain direction (*θ*) is the proportionality factor between the emitted intensity from a body at a given temperature (*T*), $L\left(\lambda ,T,\theta \right)$, and the spectral radiance (intensity) of a blackbody at the same *λ* and *T*, ${L}_{bb}\left(\lambda ,T\right)$^32^:

$$L\left(\lambda ,T,\theta \right)=\epsilon \left(\lambda ,\uptheta \right){L}_{bb}\left(\lambda ,T\right)$$

For the metasurfaces studied in this paper, the emissivity can be readily estimated taking advantage of the fact that

$$\epsilon \left(\lambda ,\theta \right)=\alpha \left(\lambda ,\theta \right)$$

where $\alpha \left(\lambda ,\theta \right)$ is the absorptivity^33^.

## Methods

### Numerical simulation

**Figure 1:**
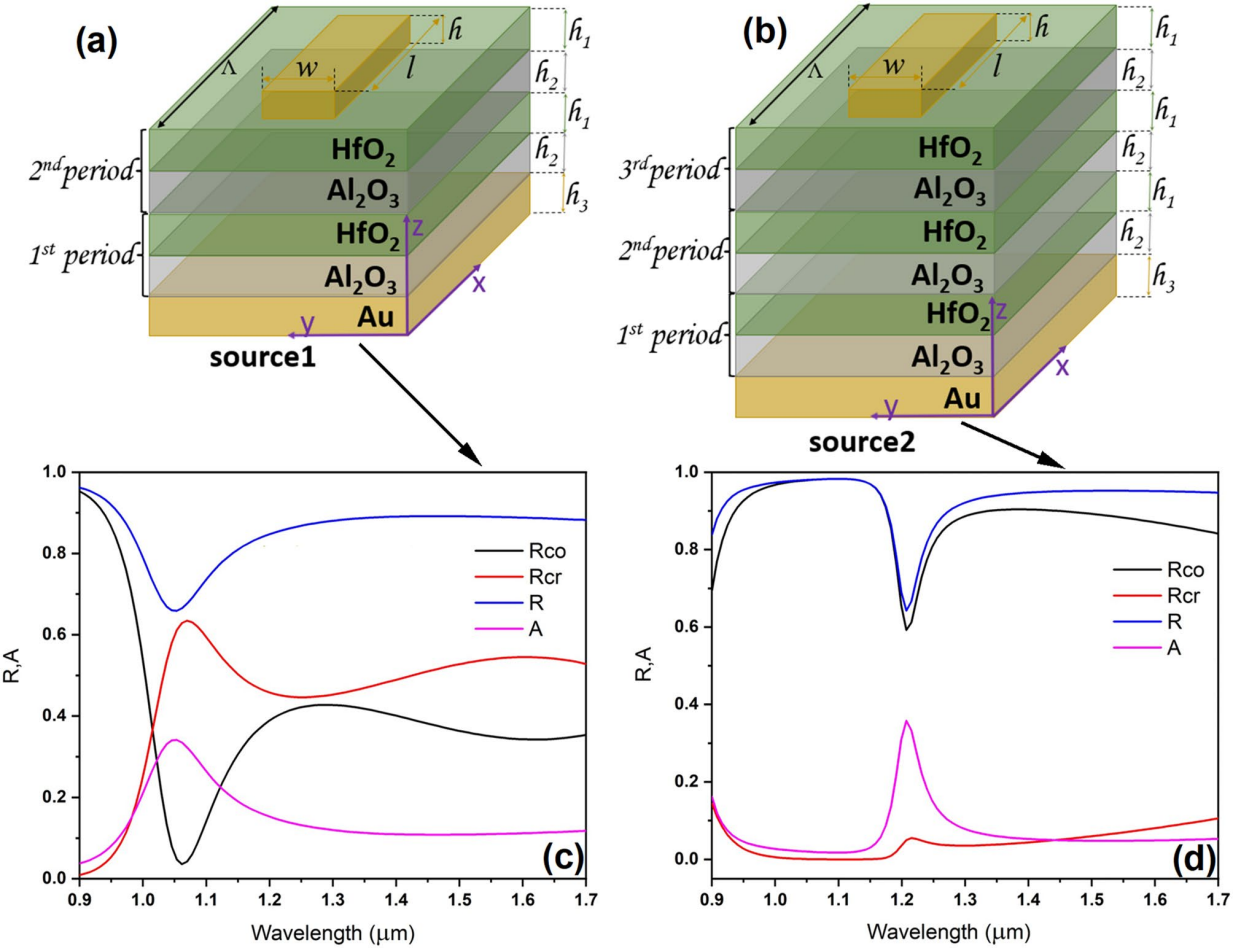
Schematic representation of the metasurfaces consisting of (**a**) 2 and (**b**) 3 HfO_2_/Al_2_O_3_ periods and the (**c**,**d**) R_cr_, R_co_, the total reflectance R and absorbance A simulation curves for normally circular polarized incident electromagnetic radiation.

**Figure 2:**
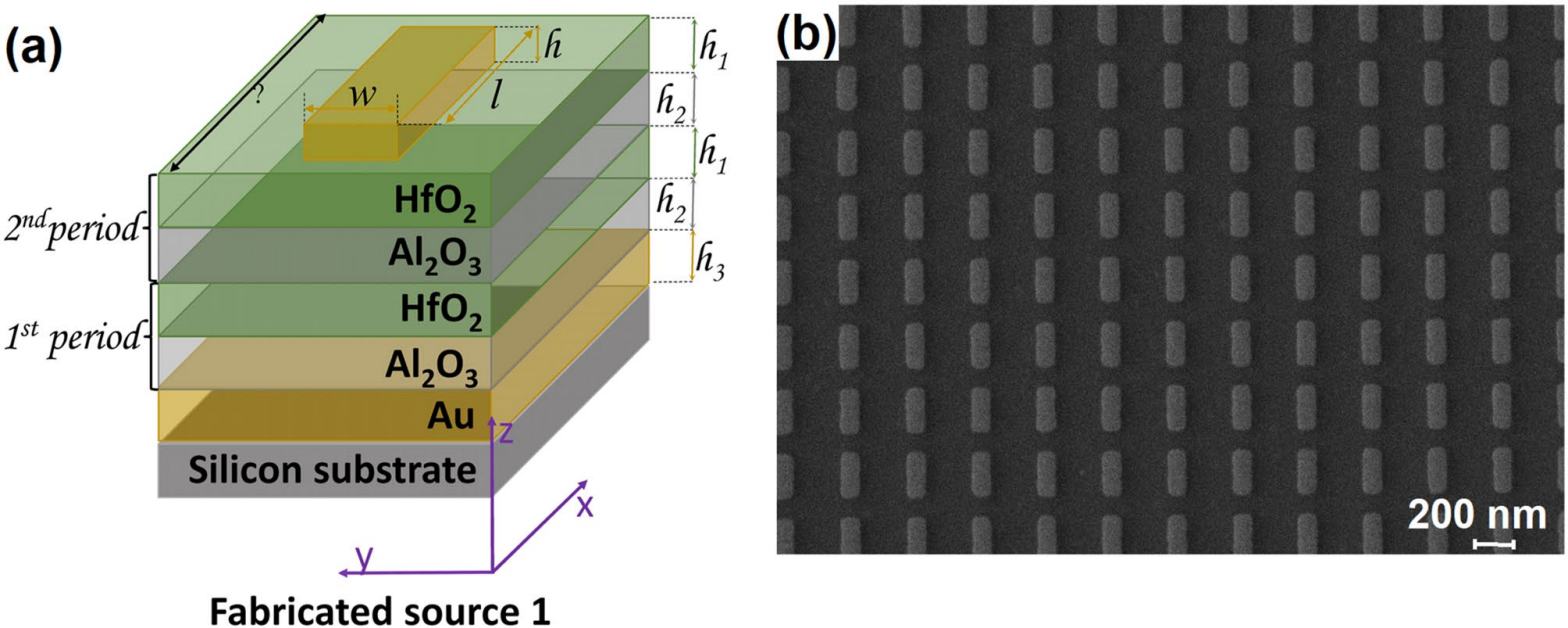
(**a**) Schematic representation and (**b**) SEM micrograph of the fabricated gold meta-atoms onto the multi-layered stack.

As specified above, since the metasurfaces we are interested in should also be configured as metalenses, they must have both significant absorbance and reflectance in NIR. More precisely, for incident circular polarized radiation, the requirement is to have a high cross-polarized reflectance (*R*_cr_), small co-polarized (*R*_co_) reflectance and high absorbance (*A*)^20^ with reasonably narrow shapes. Another desired feature would be that the metasurfaces are CMOS compatible and readily fabricated by standard technologies such as atomic layer deposition. For this reason, we studied periodic dielectric configurations consisting of alternate layers of HfO_2_ and Al_2_O_3_, which also form good interfaces. In addition, we have focused on structures with easily achievable thicknesses of different layers. This multi-layered structure was considered to be placed on a bottom metallic (Au) layer with a thickness of 50 nm (*h*_*3*_) much larger than the skin depth, of about 23 nm at a wavelength of 1300 nm, and rectangular metallic meta-atoms are patterned on top of it (see Fig. 1a,b, which show only the unit cell of the corresponding metasurface considered infinitely extended along the *x* and *y* directions). As such, the structures do not allow light transmission through them, fact verified by numerical simulation. Note that, for ease of manipulation, in practice the configuration just described was deposited on a thick doped silicon substrate (see also “Methods” and Fig. 2), the substrate having no bearing on the simulation results since, as justified above, the light does not reach the bottom layer/substrate interface.

The aim of the simulation was to identify the optimum configuration and number of layers/periods in order to fulfill the requirements of reflectance and absorbance mentioned above. The chosen structure has square unit cells with a period Λ = 400 nm, the gold meta-atoms having the following dimensions: width* w* = 100 nm, length* l* = 250 nm, and height *h* = 30 nm, while the thicknesses of the HfO_2_ and Al_2_O_3_ layers were taken as *h*_1_ = 50 nm and, respectively, *h*_2_ = 40 nm. We have used the COMSOL software to compute *R*_*cr*_, *R*_*co*_, the total reflectance *R* and the absorbance *A* of metasurfaces consisting of 2 and 3 HfO_2_/Al_2_O_3_ periods for normal incidence of circularly-polarized light, the results being illustrated in Fig. 1c and, respectively, Fig. 1d. The optical constant of Au was taken from the material database in COMSOL (in turn taken from^34^ for 0.188–1.937 µm), while the dielectric constants for HfO_2_ and Al_2_O_3_ were taken from literature: *n* = 1.879 for HfO_2_ around 1.3 µm^35^ and *n* = 1.75 for Al_2_O_3_^36^. Periodic boundary conditions were imposed along the *x* and *y* directions, and plane wave excitation was considered along the *z* direction (normal to the structure). From Fig. 1c,d it follows that the resonant wavelength as well as the spectral width of the resonance depends on the number of dielectric periods.

### Fabrication of the multi-layered configurations with metallic meta-atoms

*The fabrication of the multi-layered configuration* was carried out by atomic layer deposition (ALD) on *p* type silicon wafers (4 inches, < 100 > orientation), with a resistivity of 5–10 Ω cm and 525 µm thickness, purchased from Siegert Wafer. Prior to the ALD process, the silicon wafers were subjected to a surface cleaning treatment by exposure to UV-ozone for 15 min at 75 °C, using an UV-Ozone cleaner (Novascan Technologies, USA). A thin film of 5/50 nm of Ti/Au was deposited onto the substrate using the sputtering technique. Subsequently, an Oxford Instruments OpAL ALD reactor was used for the deposition of Al_2_O_3_ and HfO_2_ thin films using Trimethylaluminum (TMA, 99.999 + %-Al, Puratrem, USA) and Tetrakis(dimethylamino)hafnium (TDMAH, 99.99 + %-Hf, < 0.2%-Zr, Puratrem, USA) as metal organic precursors. Ultra-pure water was used as the oxidant, and ultra-high purity nitrogen (Nitrogen 6.0, 99.9999 vol. %) was used as the purge and carrier gas. The oxide films were grown in a laminar manner by alternating the ALD cycles, in a single step process; thus, films with a thickness of 40 nm of Al_2_O_3_ and 50 nm of HfO_2_ were deposited at 200 °C. The ALD deposition process was repeated for two and three times in order to obtain 2 periods of Al_2_O_3_/HfO_2_ and 3 periods of Al_2_O_3_/HfO_2_, respectively.

*The fabrication of the metallic meta-atoms onto the multi-layered stack.* The Au meta-atoms were fabricated by the electron-beam lithography process. Briefly, an electroresist (PMMA 950 k A4) spin-coated at 2000 rpm and backed on a hotplate at 160 °C, for 3 min, was used to create the template (patterns) for the rectangular meta-atoms deposition by electron beam irradiation with a dedicated e-beam equipment (RAITH e_Line), with the following parameters: acceleration voltage − 30 kV; 20 µm aperture, a beam current of 107 pA, 10 mm working distance and 100 µm × 100 µm write field. To reveal the patterns, the irradiated wafers were developed in a mixture of MIBK:IPA (1:3), for one minute, at room temperature. The metallic meta-atoms (with a thickness of 30 nm) were deposited using a highly directional e-beam evaporator (TEMESCAL FC2000) at a deposition rate of 3 A/sec. Prior to gold deposition, a thin layer of Ti was deposited onto the multi-layered stack at a deposition rate of 1 A/sec. The final structures (see Fig. 2) were revealed after the lift-off process, consisting of acetone immersion for few hours, at room temperature.

### Optical measurements

Fourier Transform Infrared spectroscopy (FTIR) measurements were performed using a JASCO FT/IR 6600 equipment in two different operation modes: (a) with a specular reflectance standard module RF-81S, scan range 1–1.7 μm with 1 cm^−1^ resolution using a TGS detector and aperture of 3.5 mm and (b) with an integrated sphere using an InGaAs detector, scan range 0.9–1.7 μm with 4 cm^−1^ resolution.

Reflection measurements at different incidence angles were performed using a variable angle spectroscopic ellipsometer (Woollam V-VASE). The *R*_*s*_ and *R*_*p*_ data, corresponding to s- and p-polarized incidence waves, were acquired from 0.3 to 1.35 μm with a 0.01 μm step, at three different angles: 35°, 60° and 75°.

## Results and discussions

This section presents the results of optical characterizations of the two samples consisting of metasurfaces made of gold meta-atoms and placed onto 2 and 3 HfO_2_/Al_2_O_3_ periods, similar with the schematic designs from Fig. 1a,b, denoted as source 1 and source 2, as well as the comparison to their numerical simulations. The plots in Fig. 1c,d were obtained for normally incident circular polarized electromagnetic radiation. However, the optical characterizations in this section were performed for linearly polarized light. As such, new simulations have been produced in order for the comparison to be relevant. On the other hand, in actual applications it would be simpler to illuminate the metasurface with linear polarized light than with circular polarized radiation. Therefore, the results in this section could be of more practical interest than those in the previous one.

**Figure 3:**
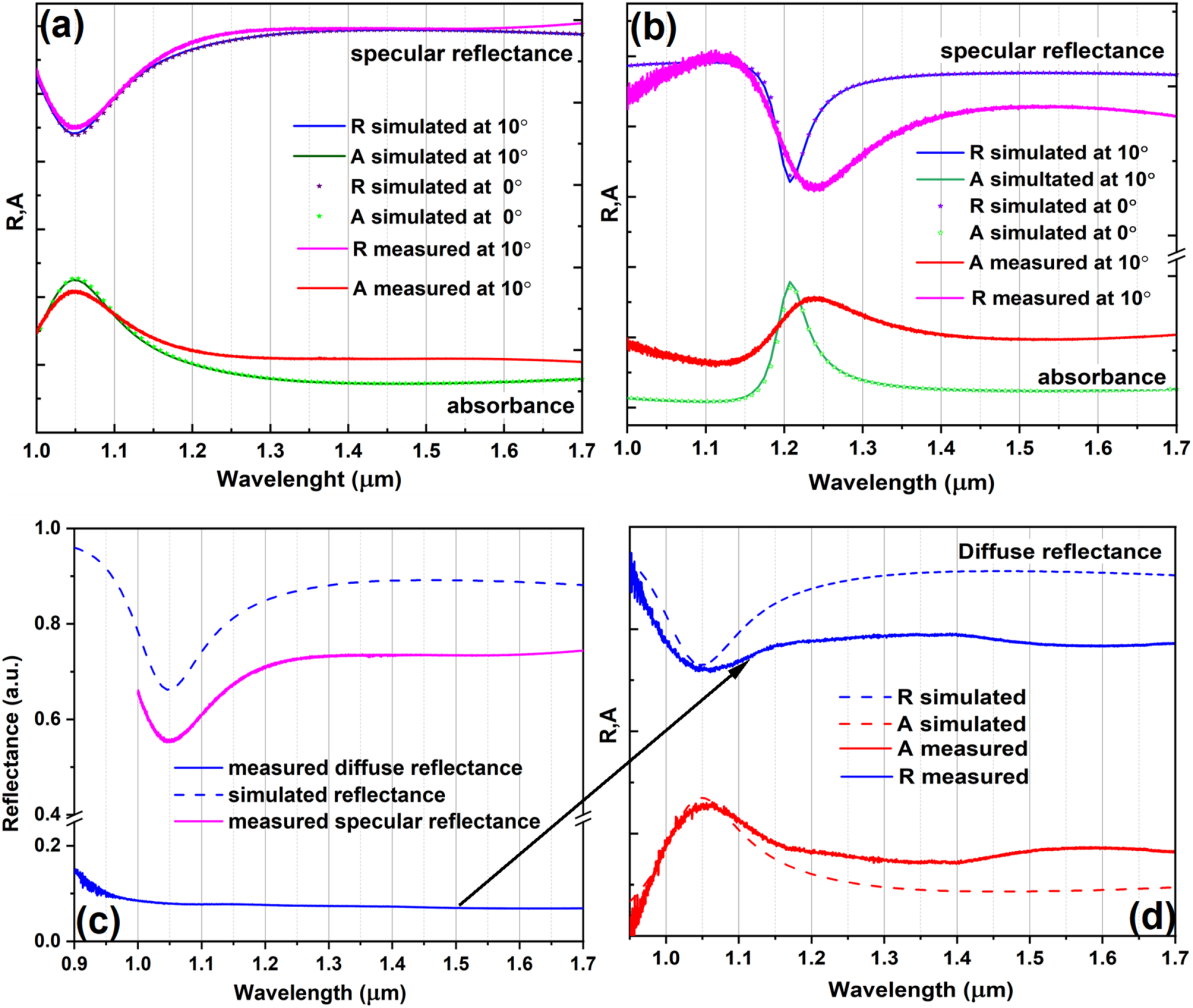
FTIR reflectance measurements: (**a**) specular reflectance measurements and the simulated data for the metasurface with 2 periods—source 1 and with (**b**) 3 periods—source 2 at different incidence angles, comparison of the: (**c**) specular and diffuse reflectance measurements with the simulated data for the metasurface with 2 periods—source 1, (**d**) normalized measured diffuse reflectance and absorbance curves with the simulated reflectance and absorbance ones.

The first measurements that we performed are FTIR measurements with a reflectance standard module RF-81S, obtaining the specular reflectance of the sample at 10° (Fig. 3a,b). From the simulation, the shapes and the position of the *A* and *R* are not modified for an incident radiation in the range 0 ÷ 30°. Moreover, since the incident radiation does not penetrate the bottom gold layer of the two sources, according to the energy conservation law:

$$\left.\begin{array}{c}T+A+R=1\\ T=0\end{array}\right\}\Rightarrow A=1-R,$$

we can perform just reflectance measurements in order to check the emissivity at room temperature.

The specular reflectance measurements performed using FTIR device are considered as preliminary tests for the numerical simulation validation. In other words, we wanted to see if we can obtain, using the specular reflectance module, a variation of *R* and *A* in the spectral region of interest, as predicted by the simulations. As we can see from Fig. 3a, the specular reflectance signal shape is similar with the reflectance curve for linearly polarized light for an incident radiation between 0 ÷ 30°. For source 2, the shape of the registered signal is slightly larger and shifted to higher wavelengths compared to the reflectance of the source obtained by numerical simulations. However, overall, the measured general spectral dependence of both reflectance and absorbance is in agreement with the simulations, possible sources of errors being due to fabrication errors which tend to become more important as the number of layers increases. Also, it should be mentioned that the registered reflectance can be considered as a relative specular reflectance because the calibration of the instrument is done to 100% *R* in our case, but the mirror used for the baseline correction does not reflect with perfect efficiency at 100%.

Since the reflectance is dependent of the material surface, we can divide the materials in two categories: materials that give specular reflection, such as polished metals, or materials with rough surfaces. Since our sources are characterized by a top layer consisting of gold meta-atoms, we perform diffuse reflectance measurements for source 1 (Fig. 3c,d) in order to see the influence of the diffuse reflectance over the total reflectance value, which is a sum of the both diffuse and specular reflections. In Fig. 3c the measured specular and diffuse reflectance curves are plotted and compared with the corresponding simulation curves for reflectance obtained using COMSOL. As we can see, the contribution of the diffuse reflectance is almost 0.1 of the total reflectance. From Fig. 3d, where the values of the measured diffuse reflectance and absorbance are normalized and compared with the simulated values, it can be seen that the shape of the reflectance and, hence, absorption curves is preserved. Thus, for further analysis, we chose to ignore the contribution of the diffuse reflectance component in measurements and to perform specular reflectance measurements using linearly polarized light at three different angles, larger than 30°, using an ellipsometer.

**Figure 4:**
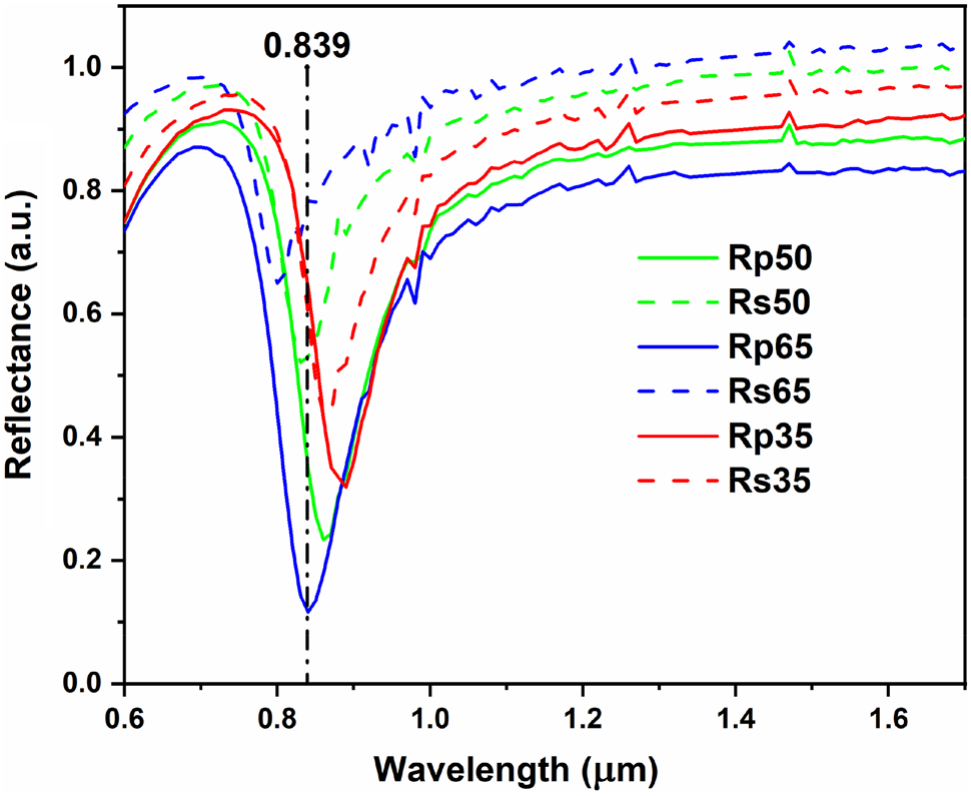
Reflectance measurements of the first structure with the top gold layer without meta-atoms.

**Figure 5:**
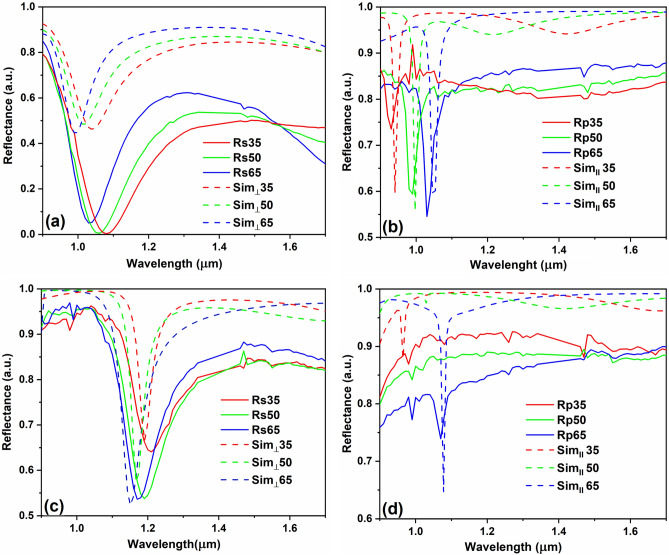
Reflectance measurements of source 1 for light polarization (**a**) perpendicular and (**b**) parallel to the incidence plan and of source 2 for light polarization (**a**) perpendicular and (**b**) parallel to the incidence plan.

In agreement with the affirmations above, we expect that the maximum specular reflection for source 1 will occur when the gold layer above the periodic HfO_2_/Al_2_O_3_ layers is continuous, thus for structures without meta-atoms. The reflectance measurements curves of the continuous gold layer deposited on top of the periodic dielectric layers at different angles are shown in Fig. 4. The measurements were performed when the angle of the polarizer is set at 0°—for the *R*_*p*_ component, when the electric fields of incident and reflected light waves oscillate within the incidence plane, and at 90°—for the *R*_*s*_ component, when the electric fields are perpendicular to the plane of incidence^37^. Also, these angles can be considered the azimuthal angles of our system. The resonance of the structure is dependent of both polarizer and incidence angles. The resonance for the *R*_*p*_ components is higher than for the *R*_*s*_ components at the same angle of incidence and the position of the resonance is shifted slightly to lower wavelengths for the case when the electric fields of the incident and reflected light are perpendicular onto the plane of incidence. Also, at wavelengths higher than 1 μm we don’t see any additional resonance, as we obtain in the case of source 1 from numerical simulation (Fig. 1c) and reflectance measurements (Fig. 5a,b). This result is to be expected since the resonant behavior of the metasurface (containing meta-atoms) is determined mainly by the periodic arrangement of the top metallic pattern.

In terms of periodic metasurfaces of period Λ, as in our case the samples denoted as source 1 and source 2, the reflectance *R*, which is connected with the absorptance *A* = 1 − *R* when the transmissivity is zero due to energy conservation, is given by the sum of the reflected light waves in all propagations orders of both *s* and *p* polarizations^21^. Therefore, the total emissivity of the sources can be expressed by the following formula: $\varepsilon =A=1-R$. For simplicity, we compare the specular reflectance measurements with the numerical simulations for both polarizations individually. From Fig. 5a–d we observe that the shape of the plots is different for the *s* and *p* polarized incident radiation. In the case of source 1, for *s* polarized incidence the measured resonance wavelength is higher that the simulated one, reaching *R* = 0 for the incidence angles of 35 and 50°. The tendency of shifting the resonance wavelength to lower values is similar to the corresponding simulated reflectances. The discrepancy between experimental data and numerical simulations can be caused by some fabrication imperfections, as for example the presence of some additional large gold nanoparticles above the meta-atoms surface. For the *p* polarized incidence wavelengths, the resonances have in general different values than for the *s*-polarized case and are shifting from lower to higher wavelengths as the incidence angles increase, contrary to the case when the components of the electric fields of the incident and reflected waves were perpendicular to the incidence plan. Thus, the actual position of the resonance wavelength can also be modified by simply changing the incidence angle, besides changing the polarization.

The general observations made for source 1 are valid also for source 2, with the only exception that for the *p* polarized incidence waves the spectral position of the resonant wavelength is more difficult to pinpoint, with the exception of the data at the 65° angle of incidence, due to a more prominent oscillatory behavior of the reflectance. The discrepancy between the simulated and measured values of the reflectance determined using the ellipsometer is lower than in the case of source 1.

**Figure 6:**
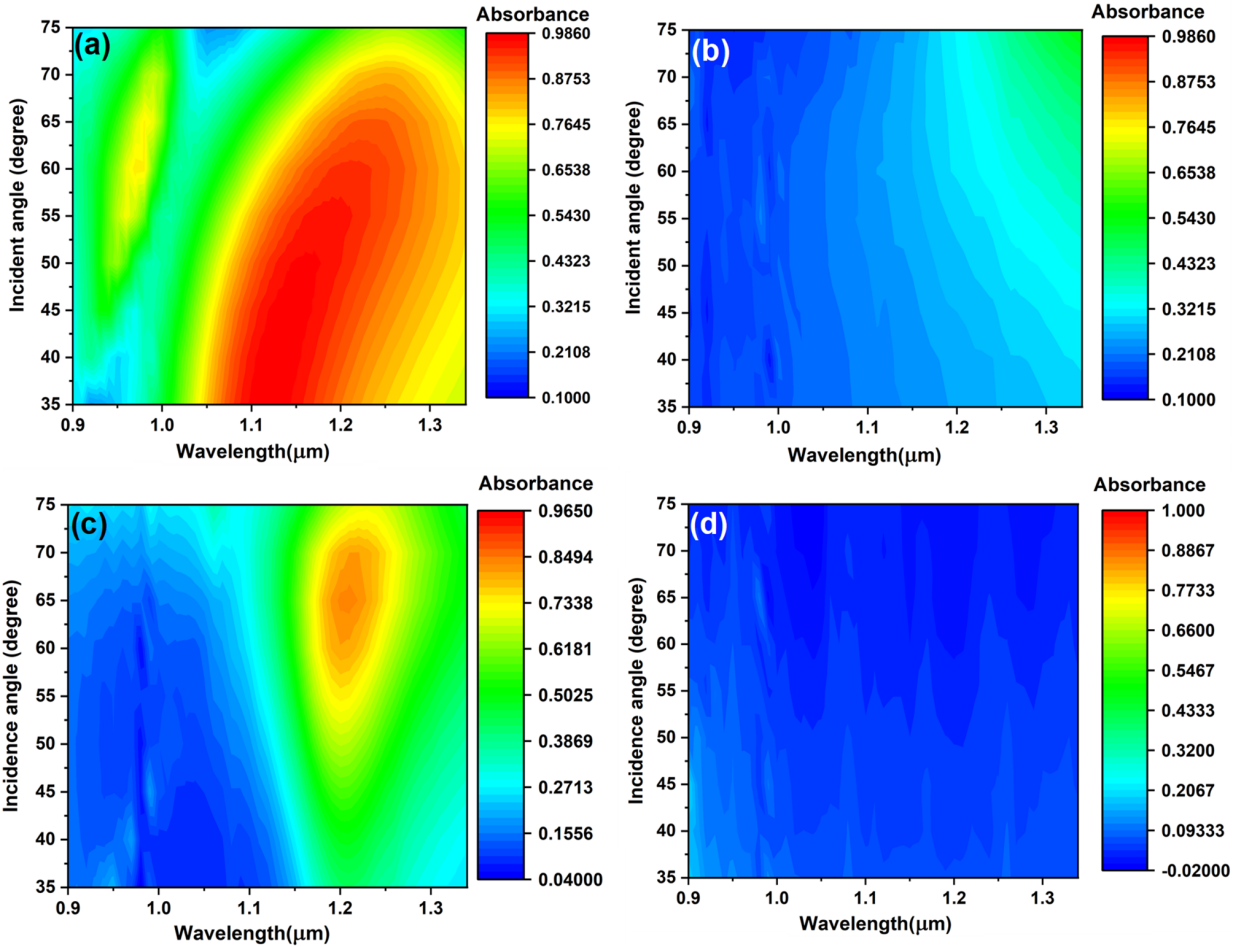
Absorbance measurements versus wavelength and incidence angle of source 1 (**a**,**b**) and source 2 (**c**,**d**) under s-polarized (**a**,**c**) and p-polarized (**b**,**d**) incidence radiation.

Further, in order to study the directivity of the emissive metasurfaces we have performed another set of reflectance measurement at different incidence angles, in which data was collected in steps of 5° and then the emissivity/absorbance was calculated as mentioned before. Figure 6 shows the *s*-polarized and *p*-polarized absorbance versus wavelengths and incidence angles for both sources. For the *s*-polarized absorbance, source 1 is characterized by a strong absorption feature observed roughly in the interval 1.1–1.25 μm and by the presence of a weak feature at lower wavelengths, that was not present in the initial reflectance measurements. The second, weaker peak can be due to incident radiation that reaches the edge of source 1 and thus contributes with a false signal to the overall absorbance. In addition, the strong absorbance peak has a wide angular distribution, i.e., poor directivity. In the case of source 2, the *s*-polarized absorbance around 1.2 μm is observed, in agreement with the previous reflectance measurements, and has a narrower distribution in both wavelengths and angles.

**Figure 7:**
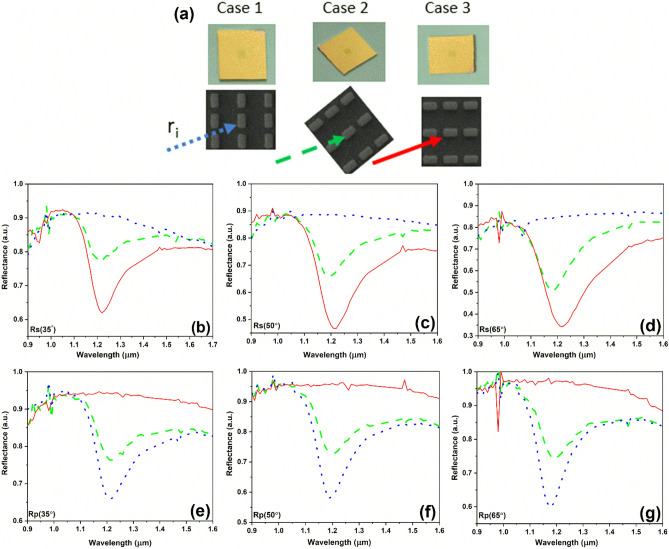
(**a**) Optical images of the source position and SEM details of the meta-atoms orientation at incident wavelength and Reflectance measurements of source 2 for light polarization perpendicular (**b**–**d**) and (**e**–**g**) parallel to the incidence plan in function of the meta-atoms position.

In the case of the *p*-polarized absorbance, no representative features are observed, fact that is in contradiction with the reflectance measurements presented in Fig. 5b,d. Since our meta-atoms have a rectangular shape, we assumed that this discrepancy between the measurement results can be due to the change in the relative position between the source and the meta-atoms at the two sets of measurements. In order to investigate this hypothesis, we performed a new series of reflectance measurements for source 2 in function of the meta-atoms position at *s*- and *p*-polarized incident radiation (Fig. 7); the corresponding SEM images of the different meta-atoms positions are shown in Fig. 7a. The experimental reflectance data corresponding to the three orientations of meta-atoms considered (and defined as case 1, case 2, and case 3 in Fig. 7a) are plotted in Fig. 7b–g with the same line type.

Analyzing Fig. 7, one can see that the orientation of the meta-atoms is very important for reflectance measurements. For example, for cases 1 and 3 of the same source 2, the reflectance values around the resonance are completely changed at both *s*- and *p*-polarized incidence waves. In other words, source 2 does not show any resonance behavior in case 1, while in case 3 it presents a resonance at around 1.2 μm for *s*-polarized incident waves (Fig. 7b–d), behavior which is opposite to the measurements for *p*-polarized incident waves, where source 2 presents resonance at 1.2 μm in case 1 (Fig. 7f,g). This resonance is slightly smaller than the corresponding one for case 3 of the *s*-polarized incident wave. Similar results were obtained for the measured reflectance at all incident angles considered (35°, 50° and 65°), as can be seen from Fig. 7. These results show that the orientation of meta-atoms is also an important parameter, besides the dimensions (widths and lengths) of the meta-atoms, and can tune the value of the reflectance, and hence of the absorbance/emittance at the resonant wavelength.

## Conclusions

Starting from a multifunctional metasurface, that could act both as metalens and thermal emitter, we have performed numerical simulations to identify the optimal conditions for such a structure to emit in NIR and to be fabricated in a cost-effective manner. Then, we have fabricated the optimized structures and characterized them optically, by measuring their reflectance as a function of wavelength, polarization and incidence angle. The emittance/absorbance can be determined from the measured reflectance spectra. Overall, the agreement between the experimental results and numerical simulations were satisfactory.

Moreover, we have shown that the resonance wavelength can be tuned by modifying the configuration of the metasurface, more precisely the number of dielectric periods, as well as the linear polarization (*s* or *p*) and the incidence angle. Also, the reflectance value at resonance depends on the orientation of the meta-atoms with respect to the direction of the incident radiation.

Our results show that metasurfaces with tunable characteristics in NIR useful for plastic detection can be obtained using standard fabrication methods and that numerical simulations are essential to design their optical properties. The designed metasurface can eventually be converted into a thermally emitting metalens, if meta-atoms in adjacent cells are suitably rotated one with respect to others, as discussed in Ref.^20^. Such a structure is beyond the scope of this paper but, for it to work, it must rely only on geometric phases imposed by the integral rotation of meta-atoms, and not on chirality-assisted phases, as in the case of the metasurfaces in Refs.^38,39^, for example.

## Data Availability

The datasets used and/or analyzed during the current study available from the corresponding author on reasonable request.
